# Useful Suggestions

**Published:** 1846-10

**Authors:** 


7. *Useful Suggestions.*—Good advice cannot be too often repeated, nor in too many forms. We have read with pleasure, therefore, the views of Dr. Hughes Bennett, as expressed in the first of a series of articles under the above title in the *Edinburgh Monthly Journal.* He sums up the arguments which he advocates as a series of suggestions for the advancement of our science, and we extract them nearly in the author's words.—*Lancet.*

"1. To encourage the idea among the profession which considers him to be the truly practical man who exercises a sound reason and judgment in the practice of medicine and surgery, based rather upon a knowledge of anatomy and physiology—morbid anatomy and pathology—than upon mere experience.

"2. To encourage the habitual use of specula, stethoscopes, pleximeters, sounds, microscopes, and every instrument, capable of bringing the products of disease under the immediate cognisance of the senses, and thus rendering diagnosis exact.

"3. To encourage the study of pathological anatomy on rational grounds—that is, by examining *all* the organs in every case—investigating into the minute structure of every morbid product—and by obtaining a chemical analysis of these, and of the blood, whenever this is practicable.

"4. To place in all hospitals connected with medical schools an officer well acquainted with morbid anatomy, and the modern means of cultivating it, whose duty it shall be to conduct the post-mortem examinations, keep a minute record of each, teach morbid anatomy to the students, and publish a yearly report.

"5. That in our public institutions the history of disease should not be recorded by young men inexperienced in observation, but should in all cases be dictated by the physician and surgeon.

"6. To extend and give greater importance to clinical instruction by introducing the system of bed-side tuition, so advantageously practised in continental universities, and by taking care that those who teach are enabled to communicate to their pupils the manual dexterity and knowledge in the use of all those instruments essential to an exact diagnosis.

"7, and lastly. To impress upon the legislature the necessity of introducing some system which will ensure the appointment to our public hospitals of well-educated physicians and surgeons, intimately acquainted with pathology and the principles of rational medicine. Otherwise, it cannot be reasonably anticipated that the extensive opportunities for observation which these institutions afford will ever be made available in advancing the healing art for the good of the community at large."—*Bulletin of Medical Science.*

